# Innominate Artery Transection via Combined Suprasternal and Intercostal Approach Prevents Tracheoinnominate Artery Fistula

**DOI:** 10.3400/avd.cr.25-00099

**Published:** 2026-01-14

**Authors:** Masahide Shichijo, Hiroyuki Morokuma, Nagi Hayashi, Takashi Teishikata, Masafumi Hiratsuka, Keiji Kamohara

**Affiliations:** Department of Thoracic and Cardiovascular Surgery, Faculty of Medicine, Saga University, Saga, Saga, Japan

**Keywords:** tracheoinnominate artery fistula, innominate artery transection, prophylactic transection

## Abstract

Tracheoinnominate artery fistula is a rare but potentially fatal complication of tracheostomy. We report the case of a 22-year-old male at high risk for tracheoinnominate artery fistula due to severe thoracic deformity. To mitigate the risk, a prophylactic transection of the innominate artery was successfully performed using a combined suprasternal and intercostal approach, thereby avoiding limb perfusion. The patient was discharged without complications. This case highlights the effectiveness of the combined approach for safe innominate artery transection in anatomically challenging cases.

## Introduction

Tracheoinnominate artery fistula (TIF) is a rare but potentially fatal complication of tracheostomy. Prophylactic transection of the innominate artery has been recommended in high-risk cases to prevent fistula formation.^1,2)^ Although median sternotomy remains the standard approach for accessing the innominate artery, the suprasternal approach has recently been reported as a less invasive alternative, potentially lowering the risk of mediastinitis.^3)^ However, in certain patients, particularly those with severe thoracic deformities, anatomical limitations may prevent access to the proximal innominate artery when employing the suprasternal approach alone. We present a case in which successful transection of the innominate artery was achieved without sternotomy by combining the suprasternal and intercostal approaches.

## Case Report

**Fig. 1:**
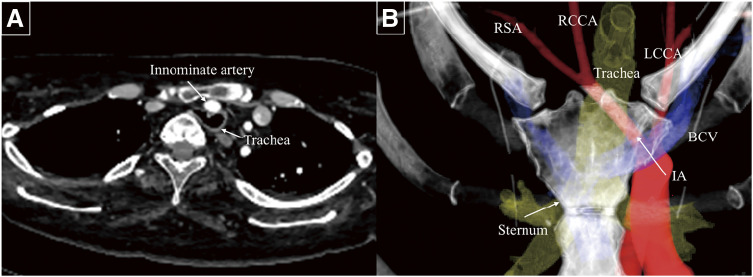
Preoperative contrast-enhanced computed tomography. (**A**) A 10-mm space between the sternum and vertebral bodies with tracheal compression from the adjacent innominate artery. (**B**) Three-dimensional computed tomography showing the tracheostomy site, sternum, and major arteries; the aortic arch and innominate artery origin were displaced leftward. LCCA: left common carotid artery; BCV: brachiocephalic vein; RCCA: right common carotid artery; RSA: right subclavian artery; IA: innominate artery

A 22-year-old male with a history of hypoxic encephalopathy and laryngotracheal separation at ages 1 and 5 years, respectively, has had a tracheal cannula in place since early childhood. Bronchoscopy revealed a pulsating elevation of the tracheal mucosa. Contrast-enhanced computed tomography (CT) demonstrated a narrowed space of 10 mm between the posterior aspect of the sternum and the vertebral body, with tracheal compression and deviation (**Fig. 1A**). Cerebral blood flow imaging revealed adequate collateral circulation via the circle of Willis. Given the high risk of TIF formation, prophylactic innominate artery transection was recommended. Owing to the close proximity of the tracheostomy stoma to the sternum, which increased the risk of mediastinitis, a suprasternal approach was initially considered. However, because the origin of the innominate artery deviated to the left side, accessing and transecting the artery at its origin using this approach alone was challenging (**Fig. 1B**). Therefore, an intercostal approach was added to allow appropriate management of the innominate artery origin.

**Fig. 2:**
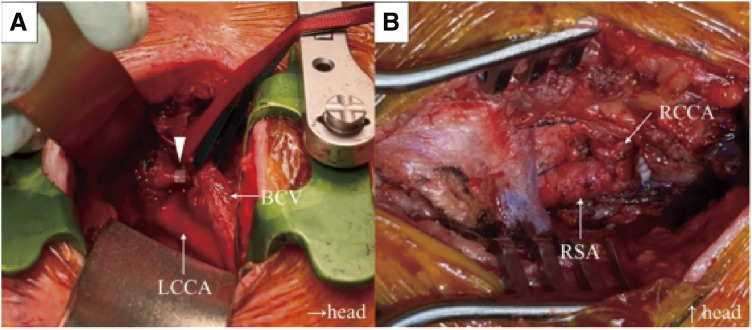
Intraoperative findings. (**A**) A skin incision was made at the level of the left first rib, and the origin of the innominate artery was ligated using a Hem-o-lok clip (Teleflex Medical, Morrisville, NC, USA) (arrowhead). (**B**) A skin incision was made above the right clavicle to expose the innominate artery, RCCA, and RSA, which were then transected. The distal end of the innominate artery was closed using 5-0 Prolene sutures (Ethicon, Somerville, NJ, USA) to preserve communication between the RCCA and RSA. LCCA: left common carotid artery; BCV: brachiocephalic vein; RCCA: right common carotid artery; RSA: right subclavian artery

The patient was placed in the supine position, and a spiral tube was inserted through the tracheostomy site under general anesthesia. Cerebral and right upper extremity perfusion were monitored using regional cerebral oxygen saturation (rSO_2_) and a right radial arterial pressure line, respectively. A skin incision was made at the level of the first left rib, and the first costal cartilage was transected at its sternal attachment. The origin of the innominate artery was exposed, and a test clamp was performed. Continuous monitoring of rSO_2_ and radial artery pressure revealed no significant decrease in cerebral or right upper limb perfusion, allowing safe ligation of the artery using 2 Hem-o-lok L clips (Teleflex Medical, Morrisville, NC, USA) (**Fig. 2A**). In the case of compromised cerebral or upper extremity perfusion, we were prepared to perform a bilateral subclavian artery bypass using an 8-mm ringed expanded polytetrafluoroethylene graft under partial clamping. However, intraoperative monitoring indicated sufficient collateral flow, thus bypass was not required. A second skin incision was made above the right clavicle to expose the innominate, right common carotid, and right subclavian arteries. The right common carotid and right subclavian arteries were clamped, and the innominate artery was transected. The distal stump was closed using 5-0 Prolene sutures (Ethicon, Somerville, NJ, USA) to preserve the continuity between the right common carotid and right subclavian arteries (**Fig. 2B**). The total operative time was 213 min, with an estimated blood loss of 103 mL.

**Fig. 3:**
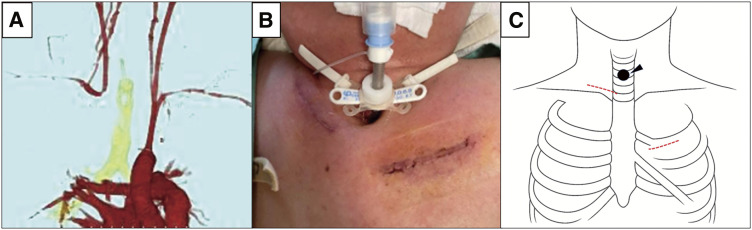
(**A**) Postoperative contrast-enhanced computed tomography scan demonstrated the absence of blood flow at the origin of the innominate artery, while the flow in the right common carotid and right subclavian arteries was preserved. (**B**) Surgical findings. The patient had a short neck, and the tracheostomy site was in close proximity to the sternum. (**C**) Surgical schema. The arrowhead indicates the tracheostomy site, and the red dashed lines denote the skin incisions (right supraclavicular and left intercostal approaches).

Postoperative CT demonstrated resolution of the proximity between the innominate artery and the trachea (**Fig. 3A**). The patient was discharged on postoperative day 6 without any postoperative complications, such as central nervous system dysfunction, right upper limb ischemia, or surgical site infections.

## Discussion

TIF is a rare but serious complication, occurring in approximately 0.1%–1.0% of cases following tracheostomy or laryngotracheal separation surgery.^4)^ According to a systematic review by Ward K et al., the overall mortality rate associated with TIF is approximately 64.5%.^5)^ Furthermore, Taechariyakul et al. reported that the mortality rate approaches 100% in untreated cases owing to massive bleeding or airway obstruction, highlighting the severity of this condition.^6)^ Therefore, prevention is essential for improving patient outcomes.

Children with severe neuromuscular disorders are particularly vulnerable to tracheal compression or narrowing caused by thoracic cage or spinal deformities. Owing to the fragility of both the vasculature and trachea, the incidence of TIF in this population increases to 4.4%–12.7%.^7)^ With recent advances in pediatric care, the number of such high-risk cases has increased, and prophylactic transection of the innominate artery has been reported in high-risk cases of fistula formation.^1,2)^ However, a consensus regarding the indications and optimal surgical strategy for this procedure has yet to be established.

Evaluating the risk factors for TIF involves clinical findings, CT imaging, and bronchoscopic assessment. Important clinical indicators include anatomic features such as a short neck or scoliosis, a history of laryngotracheal separation, and prior episodes of tracheal bleeding. CT imaging findings suggestive of high-risk cases include close proximity between the trachea and innominate artery, compression or deformation of the trachea by the artery, and a distance of <2 cm from the posterior surface of the sternum to the anterior aspect of the vertebral body.^3)^ Bronchoscopic findings such as tracheal mucosal erosion, ulceration, or poor granulation tissue formation have also been identified as risk factors,^8)^ especially in patients with a history of sentinel tracheal bleeding. In the present case, the severe physical and intellectual disabilities of the patient, combined with significant thoracic deformities causing tracheal compression, placed the patient at high risk for TIF. Therefore, prophylactic innominate artery transection was considered appropriate.

Two key considerations inform the surgical strategy for prophylactic transection of the innominate artery. The first is the surgical approach, and the second is whether to perform a simple transection or add a bypass to prevent cerebral and right upper limb ischemia.

Although full or partial median sternotomy remains the standard approach, some studies have reported the use of a suprasternal approach in selected cases where anatomical feasibility minimizes surgical invasiveness and reduces the risk of mediastinitis, particularly in prophylactic settings.^2,3)^ Contrast-enhanced 3D CT imaging is highly useful in determining the optimal surgical approach.^9)^ In the present case, a high risk of mediastinitis initially led to a plan for a suprasternal approach. However, the leftward displacement of the aortic arch and innominate artery origin precluded transection via this approach alone (**Fig. 1B**). An additional intercostal approach effectively exposed the origin of the innominate artery.

The decision between simple transection and bypass should be guided by preoperative imaging (CT or magnetic resonance imaging) to confirm the adequacy of intracranial collateral circulation. Intraoperatively, rSO_2_ monitoring assesses cerebral perfusion, while arterial lines in both radial arteries monitor upper limb perfusion. Furukawa et al. recommend adding a bypass if rSO_2_ decreases by ≥10% or if radial artery pressure drops by ≥30 mmHg during test clamping of the innominate artery.^10)^ At our institution, simple transection of the innominate artery is performed when either a decrease in rSO_2_ is ≤10% or a drop in systolic blood pressure in the right upper limb is ≤30 mmHg.

The technique is suitable for patients in whom (1) the tracheostomy site is close to the sternum, (2) the aortic arch or innominate artery origin is deviated to the left, and (3) mediastinal access via sternotomy is considered high risk. Contraindications include prior surgery or infection in the same field, or insufficient collateral cerebral circulation.

Between July 2014 and January 2025, 11 patients underwent prophylactic innominate artery transection at our institution. Of these, 10 met the criteria for simple transection and experienced no postoperative neurological deterioration or signs of right upper limb ischemia.

## Conclusions

In patients at high risk of TIF, prophylactic innominate artery transection can be safely performed without sternotomy by combining suprasternal and intercostal approaches when the origin of the artery is not adequately accessible when employing the suprasternal approach alone.

## Declarations

### Data availability statement

All data supporting the findings of this study are included in the article.

## References

[1] Hasegawa T, Oshima Y, Hisamatsu C, et al. Innominate artery compression of the trachea in patients with neurological or neuromuscular disorders. Eur J Cardiothorac Surg 2014; 45: 305–11. 23868953 10.1093/ejcts/ezt346

[2] Suzuki K, Fujishiro J, Ichijo C, et al. Prophylactic innominate artery transection to prevent tracheoinnominate artery fistula: a retrospective review of single institution experience. Pediatr Surg Int 2021; 37: 267–73. 33388953 10.1007/s00383-020-04792-z

[3] Fujimoto Y, Hirose K, Ota N, et al. Suprasternal approach for impending tracheo-innominate artery fistula. Gen Thorac Cardiovasc Surg 2010; 58: 480–4. 20859729 10.1007/s11748-010-0598-7

[4] Jones JW, Reynolds M, Hewitt RL, et al. Tracheo-innominate artery erosion: successful surgical management of a devastating complication. Ann Surg 1976; 184: 194–204. 782389 10.1097/00000658-197608000-00011 PMC1344431

[5] Ward K, Hinchman-Dominguez D, Stokes L, et al. A systematic review of mortality associations in patients who develop tracheoinnominate artery fistula following tracheostomy. Am Surg 2024; 90: 1648–56. 38217444 10.1177/00031348241227211

[6] Taechariyakul T, Keller FS, Jahangiri Y. Endovascular treatment of tracheoinnominate artery fistula: case report and literature review with pooled cohort analysis. Semin Thorac Cardiovasc Surg 2020; 32: 77–84. 31425754 10.1053/j.semtcvs.2019.08.006

[7] Mizuno Y, Ukaji K. Complications of tracheostomy in patient with severe motor and intellectual disabilities. No To Hattatsu 2005; 37: 517–21. (in Japanese) 16296357

[8] Mizumoto M, Uchida T, Kuroda Y, et al. Preventive innominate artery transection for a high-risk case of tracheo-innominate artery fistula. Jpn J Cardiovasc Surg 2021; 50: 337–41. (in Japanese)

[9] Hasegawa T, Zaima A, Hisamatsu C, et al. Minimally invasive innominate artery transection for tracheomalacia using 3-dimensional multidetector-row computed tomographic angiography: report of a case. J Pediatr Surg 2010; 45: E1–4. 10.1016/j.jpedsurg.2010.04.003 20638508

[10] Furukawa K, Kamohara K, Itoh M, et al. Operative technique for tracheo-innominate artery fistula repair. J Vasc Surg 2014; 59: 1163–7. 24239114 10.1016/j.jvs.2013.09.013

